# Plasma Proteomic Dynamics Preceding Glaucoma Reveal a 15-Year Pre-Diagnostic Window: Causal Insights and Predictive Utility in 45,850 Participants

**DOI:** 10.1167/iovs.67.3.14

**Published:** 2026-03-06

**Authors:** Yingke Zhao, Jiawen Wu, Chenchen Li, Yun Cheng, Qian Li, Jianing Wu, Zhongmou Sun, Shenghai Zhang, Jihong Wu

**Affiliations:** 1 Department of Ophthalmology, Eye & ENT Hospital, College of Medicine, Fudan University, Shanghai, China; 2 Shanghai Key Laboratory of Visual Impairment and Restoration, Science and Technology Commission of Shanghai Municipality, Shanghai, China; 3 Key Laboratory of Myopia (Fudan University), Chinese Academy of Medical Sciences, National Health Commission, Shanghai, China; 4 Shanghai Medical College, Fudan University, Shanghai, China; 5 BronxCare Health System Department of Ophthalmology, New York, United States

**Keywords:** glaucoma, plasma protein, biomarkers, early detection

## Abstract

**Purpose:**

To characterize the temporal dynamics of plasma proteomic changes preceding glaucoma diagnosis, identify causal proteins, and evaluate their predictive utility for early detection.

**Methods:**

We conducted a prospective cohort study of 45,850 UK Biobank participants without baseline glaucoma, followed for a median of 16.26 years. Plasma levels of 2920 proteins were measured using the Olink Explore 3072 platform. Cox proportional hazards models identified proteins associated with incident glaucoma. Mendelian randomization (MR) using cis- protein quantitative trait loci established causal relationships. Temporal trajectories were modeled using LOESS regression, and time-stratified machine learning models were developed to assess predictive performance.

**Results:**

During follow-up, 977 incident glaucoma cases were identified. After comprehensive adjustment, 136 proteins were significantly associated with glaucoma risk (false discovery rate < 0.05), with EDA2R showing the strongest association (hazard ratio [HR] = 1.21, 95% confidence interval [CI] = 1.16–1.25, *P* = 2.99 × 10^−17^). MR analysis identified four causal proteins: LTBP2 (odds ratio [OR] = 1.52, 95% CI = 1.36–1.71, *P* = 1.07 × 10^−12^), NRP2 (OR = 0.85, 95% CI = 0.78-0.92, *P* = 4.73 × 10^−5^), TNFSF13, and HAVCR1—implicating extracellular matrix remodeling and immune dysregulation in disease pathogenesis. Proteomic dysregulation commenced 12 to 15 years before clinical diagnosis, with three distinct temporal patterns identified. Time-stratified predictive models incorporating these signatures achieved an area under the curve of 0.803 (95% CI = 0.772-0.837) for up to two-year prediction, a 14.65% improvement over demographic models.

**Conclusions:**

This study reveals a 15-year window of detectable plasma proteomic dysregulation preceding glaucoma diagnosis. The identified causal proteins, particularly LTBP2, provide mechanistic insights and represent potential therapeutic targets. The strong predictive performance demonstrates the translational potential of these findings for risk-stratified screening.

Glaucoma represents the leading cause of irreversible blindness globally and is expected to affect more than 112 million individuals by 2040, resulting in a medical treatment burden of $14.6 billion.^1^^,^^2^ The asymptomatic characteristics of early-stage glaucoma complicate the diagnosis and monitoring. Infrequent testing fails to catch subtle pathology, highlighting the necessity for advanced approaches to identify at-risk individuals before irreversible damage occurs.^3^ Although glaucoma bears a strong genetic foundation, numerous genome-wide association studies have identified more than 200 genetic loci associated with the disease.^4^^,^^5^ Only 9% to 17% of cases can be explained by these polymorphisms,^6^ because most identified single nucleotide polymorphisms (SNPs) are located in non-coding regions, and their functional implications on glaucoma pathophysiology remain ambiguous, limiting clinical utility for risk prediction and therapeutic target identification.

Proteomic signatures associated with genetic variation and clinical manifestation indicate the interaction among genetic, environmental, and lifestyle factors. Consequently, the proteome serves as an essential tool for early diagnosis and for elucidating disease progression. However, limitations in current literature have impeded the translation of these findings. First, traditional clinical studies have been constrained by small sample sizes, typically fewer than 500 participants, which compromises the statistical power and reliability of the findings.^7^^,^^8^ Although recent large-scale phenome-wide association studies in biobanks have overcome sample size constraints, these broad scans often lack the granularity required for specific ocular conditions. They typically analyze glaucoma as a binary outcome based on baseline data, thereby overlooking clinical subtypes and the dynamic temporal trajectories of biomarkers. Second, cross-sectional designs are prone to reverse causation bias and are insufficient for capturing protein patterns during disease development. Third, existing studies assume that particular proteins act as indicators of glaucoma risk across temporal contexts while ignoring the complexities of disease progression. Furthermore, numerous investigations have focused on local ocular specimens, such as tear film, aqueous humor, or vitreous fluid, which reflect localized pathogenic changes.^9^ Nevertheless, the invasive collection procedures and limited quantities have impeded compliance with these procedures.

To address these limitations, we conducted a comprehensive plasma proteomic analysis of 45,850 UK Biobank participants with prospective follow-up extending to 16.26 years. We aimed to (1) characterize the temporal dynamics of proteomic changes preceding glaucoma diagnosis, (2) identify causal proteins through Mendelian randomization, and (3) evaluate the predictive utility of time-stratified proteomic signatures. This large-scale prospective design provides sufficient statistical power to establish temporal relationships between protein alterations and disease onset (Fig. 1), offering mechanistic insights into glaucoma pathogenesis and laying the foundation for early intervention strategies.

**Figure 1.**
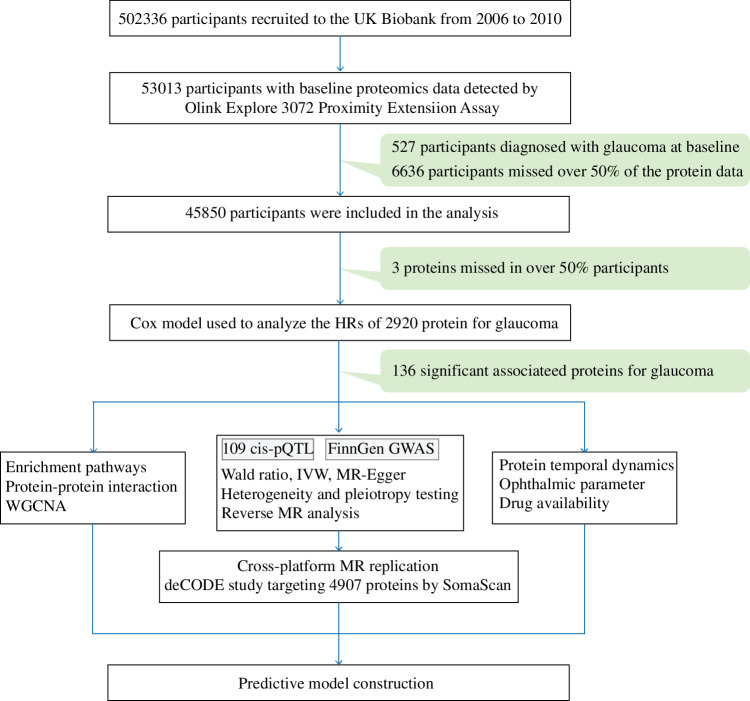
Schematic overview of the study design.

## Material and Methods

### Study Design and Participants

This prospective cohort study was sourced from the UK Biobank, an open-access biomedical database that recruited more than 500,000 participants from 22 assessment sites around the United Kingdom, documenting significant health-related data and medical records. We limited the sample to individuals without a history of glaucoma and with accessible proteome data, resulting in a total of 53,013 participants. To minimize potential bias from population stratification and genetic heterogeneity, the study population was restricted to participants of White European descent. The research received approval from the North West Multi-center Research Ethics Committee (reference number: 21/NW/0157) and was executed in compliance with the Declaration of Helsinki. All participants provided written informed consent. The research received approval from the UK Biobank project under application number 360934.

### Plasma Proteomics Tests

Out of 500,000 UKB participants, plasma samples were taken from over 50,000 individuals at the time of recruiting. Blood samples were collected from each participant in EDTA tubes. Plasma was extracted via centrifugation at 2500*g* for 10 minutes at 4°C and subsequently stored at −80°C. Between 2021 and 2022, the UKB-PPP consortium performed proteomic profiling on these baseline samples using the Olink Explore 3072 proximity extension assay. This analysis included approximately 54,000 UK Biobank participants^10^ and encompassed four panels: Cardiometabolic, Inflammation, Neurology and Oncology. The Olink protein expression data was provided as Normalized Protein eXpression values on a log_2_ scale. Details on sample selection, processing, and quality control have been documented online and addressed in prior publications.^11^ After the exclusion of proteins with over 50% missing data, 2920 proteins are selected for further study.

### Outcome Definitions

Diagnostic data were associated with many electronic health records, including hospital diagnosis data, death registry records, primary care information, and self-reported data. The primary outcome was the incidence of glaucoma, as delineated by the International Classification of Diseases, 10^th^ revision (ICD-10) code H40. Data regarding the first occurrence (Field 131186) and touchscreen questionnaire (Field 4689) were extracted to ascertain the diagnostic date. All subjects diagnosed with glaucoma at or prior to baseline enrollment were excluded (527 instances). The follow-up commences from enrollment (Field 53) until the earliest recorded date of glaucoma diagnosis, death, or the censoring data (i.e., September 15, 2024). Participants who exited the project were excluded from the study.

### Assessment of Covariates

We implemented a covariate selection framework guided by two principles: (1) controlling for pre-analytical and technical factors that influence plasma protein concentrations and (2) controlling for disease confounders without inducing collider bias or over-adjustment. First, to address technical variability in high-throughput proteomics, we adjusted for batch, assessment center, season of blood draw (to account for seasonal fluctuations in inflammatory markers), and fasting length (to control for metabolic status influence on protein levels). The top ten genetic principal components were included to account for ancestry (proxying for self-reported race) and heritable risk (addressing the lack of complete family history data), as well as to control for residual population stratification. Second, adjustments were done for demographic factors, including age, sex, body mass index, and the Townsend deprivation index, as well as possible confounders such as smoking status and alcohol consumption. Family history of glaucoma was not included as a covariate because it is not a selectable option in the standard UK Biobank touchscreen questionnaires, resulting in incomplete data coverage. Finally, we deliberately excluded intraocular pressure (IOP) and corneal biomechanical parameters (corneal hysteresis and corneal resistance factor) from the primary multivariable models. Although these are key risk factors for glaucoma, valid measurements were available for only a small subset of the proteomic cohort (*n* = 9422, ∼17% coverage). Including these variables would have necessitated a complete-case analysis, resulting in the exclusion of over 80% of participants and a significant loss of statistical power. To address this, we instead perform sensitivity analyses within the sub-cohort containing valid ocular measurements to verify that proteomic associations remained independent of IOP.

### Statistical Analysis

Cox proportional hazards regression models were employed to evaluate the relationships between plasma proteins and the onset of glaucoma. The model was initially uncorrected for covariates. Model 2 was subsequently modified for the aforementioned technical parameters. Model 3 was comprehensively adjusted for supplementary demographic data and confounding factors. The analysis was conducted on the entire cohort and at two distinct follow-up intervals to assess potential. In sensitivity analysis, we conducted the same association analyses as previously, additionally removing those who got glaucoma within two years post-baseline. A sensitivity analysis was conducted, matching 10 controls to each glaucoma patient based on age and sex, to exclude the influence of imbalanced controls. To address potential residual confounding from systemic vascular risk factors, we performed an additional sensitivity analysis by further adjusting Model 3 for systolic and diastolic blood pressure. The Benjamini-Hochberg method was used to regulate the false discovery rate in all analyses.

### Enrichment Analysis

The clusterProfiler (version 4.10.1) in the R environment (version 4.3.1) was used to make gene ontology (GO) annotations on biological processes. Analyses were adjusted for multiple testing with the Benjamini-Hochberg method.

### Protein-Protein Interactions and WGCNA Analysis

The glaucoma-associated protein underwent network analysis with STRING v.12, using a default interaction confidence threshold of 0.6. Weighted Gene Co-expression Network Analysis (WGCNA) was conducted to establish protein co-expression networks. Initially, expression data from glaucoma-related proteins underwent quality control and variance filtering to eliminate low-quality samples and low-variance proteins. The appropriate soft-thresholding parameter was determined using the pick Soft Threshold function, subsequently followed by network construction and module detection via block-wise analysis. Modules featuring an unsigned topology, a minimum module size of 8, and a merge cut height of 0.25. Module genes were computed and connected with the glaucoma disease state by Pearson correlation analysis to find disease-associated protein modules. Notable modules (*P* < 0.05) underwent functional enrichment analysis, including GO terms and Kyoto Encyclopedia of Genes and Genomes pathway analysis using clusterProfiler.

### MR Analysis

To investigate causal relationships between glaucoma-related proteins and the risk of glaucoma, we performed a two-sample Mendelian randomization analysis using protein quantitative trait loci from the UK Biobank and glaucoma genome-wide association study (GWAS) summary statistics from FinnGen (R12). We limited instrumental variables to cis-SNPs (within 1 Mb upstream and downstream from the gene encoding the protein) that attained genome-wide significance (*P* < 5 × 10^−^^8^) and were independent of linkage disequilibrium (*r*^2^ < 0.1 within a 500 kb distance using the 1000 Genomes European reference panel). Associations between protein and glaucoma were examined using the TwoSampleMR package, with method selection determined by the quantity of available instruments: Wald ratio for individual SNPs and inverse-variance weighted, MR-Egger regression, weighted median, and penalized weighted median methods for multiple SNPs. Sensitivity analyses encompassed heterogeneity evaluation by Cochran's Q test and horizontal pleiotropy assessment with the MR-Egger intercept test for proteins with three or more instruments. To corroborate important findings, we conducted replication analysis with independent pQTL data from the deCODE genetics study,^5^ integrated with the identical FinnGen glaucoma GWAS summary statistics.

### Modeling the Trajectory of Plasma Protein Fluctuations and Clustering

We used the interval between blood collection and glaucoma diagnosis as a surrogate for glaucoma progression. The trajectories of the 136 plasma proteins linked to the incidence of glaucoma were modeled as a function of follow-up years. To alleviate the effects of interindividual variability on protein concentration, up to 10 controls were matched for each case based on age and sex. Protein levels were normalized as Z-scores in relation to matched controls: Z = (case_value − control_mean)/control_SD. Protein trajectories preceding diagnosis were analyzed using LOESS regression to identify nonlinear temporal trends. Proteins exhibiting analogous temporal trajectories were found using hierarchical clustering employing correlation-based distance measures.^12^

### Temporal Stratification Prediction Model

Four temporal intervals were established: ultra-short-term (0–2 years), short-term (2–5 years), medium-term (5–10 years), and long-term (>10 years). Cases comprised participants who developed glaucoma throughout each time period, whereas controls consist of individuals with sufficient follow-up duration. The case-to-control ratio was consistently maintained at 1:4 across all investigations. Protein biomarkers were identified by recursive feature elimination with random forest. We optimized them individually for each time interval. Demographic characteristics (age, sex, and polygenic risk scores) and engineering traits, including protein ratios, interaction terms, and module statistics, were incorporated. Various modeling methodologies were utilized according to sample size. Ensemble voting classifiers were employed to elucidate intricate, non-linear correlations in ultra-short-term predictions. LightGBM with tuned hyperparameters (400 estimators, a learning rate of 0.04, and a maximum depth of 6) was used for short-term models, whereas logistic regression was used for medium- and long-term models to mitigate overfitting because of limited sample numbers. Discrimination was evaluated by fivefold stratified cross-validation, using the area under the ROC curve (AUC) as the primary metric of discrimination. Performance was reported using means and 95% confidence intervals. DeLong tests revealed substantial differences in AUC among the models.

### Ophthalmic Evaluation

In the UK Biobank enhancement effort, extensive ophthalmic evaluations were performed, including IOP measurement and optical coherence tomography (OCT) imaging. IOP was assessed in 115,000 participants using the Ocular Response Analyzer noncontact tonometer. It is important to note that although anatomical central corneal thickness was not directly measured in this cohort, the Ocular Response Analyzer device provides corneal hysteresis and corneal resistance factor as measures of corneal biomechanics. Furthermore, the device records Corneal-Compensated IOP (IOPcc), a mathematically derived index designed to minimize the influence of corneal properties on pressure readings. Both IOPcc and Goldmann-correlated IOP were recorded. Participants with recent ocular surgery, ocular infections, or prior glaucoma therapy were excluded from IOP assessments. For those using IOP-lowering medications, pretreatment values were calculated by dividing the measured IOP by 0.7. Macular spectral-domain OCT imaging was conducted on about 65,000 participants with the Topcon 3D OCT1000 Mark II with 6 × 6 mm² macular volume scans. The Topcon Advanced Boundary Segmentation method (version 1.6.1.1) was used for retinal layer segmentation.^13^ Stringent quality control eliminated OCT images with quality scores below 40 and the lowest 10% as identified by ILM indications.

### Protein-Ophthalmic Parameters Association

Following quality control processes, correlations between glaucoma-related proteins and ocular parameters were examined utilizing linear regression models. For the IOP analysis, 9442 people with high-quality data were included, with participant-level IOP computed as the average of both eyes when accessible, or single-eye values when not. The OCT parameter correlations involved people with high-quality retinal imaging. Linear regression models were constructed for each protein-IOP and protein-OCT parameter combination, controlling for pertinent factors. Corrections for multiple testing were implemented via both Bonferroni and false discovery rate methods, whereas model performance was assessed through *R*^2^ values and normalized regression coefficients.

### Evaluation of Drug Availability

We evaluated the enrichment of glaucoma-related proteins obtained from MR analysis within ICD10 illness categories using GREP (Genome for REPositioning) software.^14^ GREP consolidates drug-target data from DrugBank and the Therapeutic Target Database. Given that only TNFSF13B was present in the GREP database (totaling 2,028 genes), we augmented the study with medication information sourced from literature for the remaining three genes from DrugBank, ClinicalTrials.gov, and publications from the past five years. Fisher's exact test was used to compute enrichment odds ratios and *P* values for each ICD-10 category. The Benjamini-Hochberg method was employed to apply false discovery rate (FDR) correction. A composite priority score was computed, integrating evidence level, Food and Drug Administration approval status, and statistical significance to rank drug repositioning prospects.

## Results

### Characteristics of the Cohort

Our study comprised 45,850 participants with no previous history of glaucoma at baseline. The median age is 58 years (IQR 50–64 years), with 54.1% of participants being female and 93.1% identifying as white. The baseline characteristics are outlined in the Table. Over a median follow-up period of 16.26 years, 977 new glaucoma cases were documented. Participants who developed glaucoma were older at baseline and exhibited a greater propensity for daily alcohol intake compared to those who remained glaucoma-free (both *P* < 0.001).

**Table.** Demographic and Clinical Characteristics of UKB Participants Included in Our Study

Feature	Overall (*N* = 45850)	Cases (*n* = 977)	Controls (*n* = 44873)	*P* Value	Test
Age (years), median (IQR)	58.0 (50.0-64.0)	63 (58.0-66.0)	58.0 (50.0-63.0)	<0.001	Nonnorm
Sex					
Female	24,821 (54.1%)	525 (53.7%)	24,296 (54.1%)		
Male	21,029 (45.9%)	452 (46.3%)	20,577 (45.9%)		
Ethnicity					
White	42,669 (93.1%)	887 (90.8%)	41,782 (93.1%)	<0.001	
Asian	1096 (2.4%)	23 (2.4%)	1073 (2.4%)		
Black	1211 (2.6%)	48 (4.9%)	1163 (2.6%)		
Others	874 (1.9%)	19 (1.9%)	855 (1.9%)		
TDI, median (IQR)	−2.0 (−3.6 to 0.8)	−1.9 (−3.5 to 1.2)	−2.0 (−3.6 to 0.8)	0.053	Nonnorm
BMI, median (IQR)	26.8 (24.2–29.9)	27.0 (24.4-30.1)	26.8 (24.2-29.9)	0.05	Nonnorm
Smoking status					
Current	4895 (10.7%)	86 (8.8%)	4809 (10.7%)	0.03	
Never	24,791 (54.1%)	495 (50.7%)	24,296 (54.1%)		
Previous	15,951 (34.8%)	389 (39.8%)	15,562 (34.7%)		
Alcohol intake frequency					
Daily or almost daily	9252 (20.2%)	208 (21.3%)	9044 (20.2%)	0.001	
Three or four times a week	10,263 (22.4%)	217 (22.2%)	10,046 (22.4%)		
Once or twice a week	11,880 (25.9%)	213 (21.8%)	11,667 (26.0%)		
One to three times a month	4961 (10.8%)	96 (9.8%)	4865 (10.8%)		
Special occasions only	5420 (11.8%)	147 (15.0%)	5273 (11.8%)		
Never	3975 (8.7%)	92 (9.4%)	3883 (8.7%)		
Follow-up (years), median (IQR)	16.26 (15.59, 17.0)	8.82 (5.01, 11.2)	16.31 (15.39, 16.76)	<0.001	Nonnorm

### Proteomic Correlations With Glaucoma Risk

**Figure 2.**
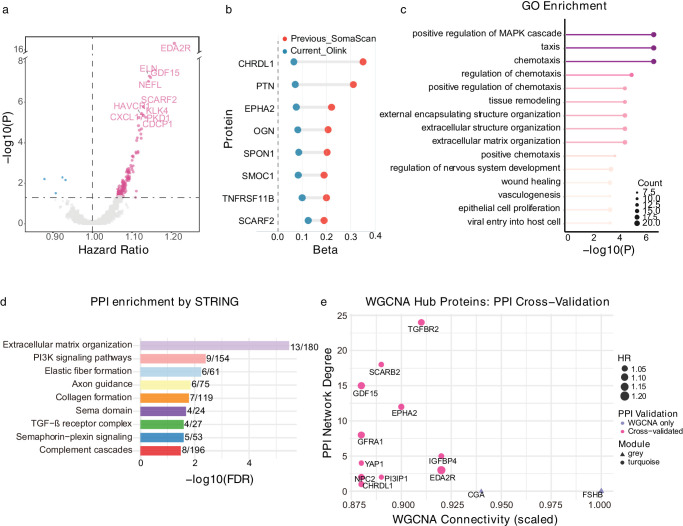
**Proteomic associations with glaucoma risk and functional characterization.** **(a)** Volcano plot displaying the association between plasma protein and glaucoma risk over the full follow-up period. The x-axis represents HR and the y-axis shows −log10 (*P* values). *Dashed lines* indicate HR = 1.0 (vertical) and statistical significance threshold (horizontal). Key proteins with strongest associations are labeled. **(b)** Cross-platform validation glaucoma-associated proteins in previous study (*red*) and current study (*blue*). **(c)** GO enrichment analysis for the 136 glaucoma-associated proteins. Biological processes are ranked by statistical significance (−log10 *P* value). **(d)** PPI network enrichment analysis using STRING database. Horizontal bars showed enriched functional cluster with the number of glaucoma-associated proteins versus total proteins in each pathway. Statistical significance is represented by −log10(FDR) values. **(e)** Cross-validation of hub proteins identified through WGCNA with PPI network analysis. The scatter plot shows WGCNA connectivity (x-axis) versus PPI network degree (y-axis). Dot sizes represent HRs, colors indicate WGCNA-only or cross-validated, and shapes distinguish WGCNA modules (*gray, turquoise*).

Cox proportional hazard models were used to study the links between levels of plasma protein and the development of glaucoma in 2920 plasma proteins measured via the Olink platform (Supplementary Table S1). In univariate analysis, 523 proteins had significant relationships with glaucoma onset (FDR < 0.05). After adjusting for variations in batch, assessment center, season, and fasting period at blood collection, as well as the top 10 genetic principal components, 451 proteins maintained statistical significance. The modified model, incorporating demographic and lifestyle variables, identified 136 proteins strongly linked to the beginning of glaucoma (FDR < 0.05) (Fig. 2a; Supplementary Table S2). Notable associations were observed for EDA2R (HR = 1.21 [1.16–1.25], *P* = 2.99 × 10^−17^), ELN (HR = 1.14 [1.10–1.19], *P* = 4.12 × 10^−^^8^), GDF15 (HR = 1.15 [1.10–1.19], *P* = 6.83 × 10^−8^), and neurofilament light polypeptide (NEFL) (HR = 1.14 [1.10–1.19], *P* = 9.17 × 10^−8^). Multiple sensitivity analyses, including removal of early cases, matched case-control analysis, cross-platform validation, confirmed our findings (Fig. 2b, Supplementary Tables S3–S5). Notably, in a sensitivity analysis additionally adjusting for systolic blood pressure, the results remained highly robust, with 264 proteins retaining statistical significance (Supplementary Table S29).

A time-stratified analysis identified notable temporal patterns. To balance statistical power and capture distinct phases of disease progression, we stratified the follow-up period at 10 years, which approximates the median time to diagnosis in our incident cases (8.82 years). Twenty-five proteins were linked to short-term events (within 10 years), with EDA2R being the most prominent (HR = 1.22 [1.15–1.30], *P* = 1.96 × 10^−6^), whereas no proteins showed significant long-term connections beyond 10 years. This observation indicates that plasma proteome indicators are more distinguishable and demonstrate higher signal-to-noise ratios as disease begins (Supplementary Fig. S1; Supplementary Tables S2, S6, and S7); hence, enhancing our subsequent time-stratified predictive modeling approach.

### Biological Functions of Proteins Associated With Glaucoma

To understanding how the 136 proteins related to glaucoma, we performed detailed analyses to explore their functions and connections. The Gene Ontology analysis identified three predominant biological processes: chemotaxis and taxis (20/136 proteins, *P* = 1.61 × 10^−10^), positive regulation of MAPK cascade (20/136 proteins, *P* = 2.02 × 10^−^^10^), and extracellular matrix organization (14/136 proteins, *P* = 8.43 × 10^−^^8^) (Fig. 2c; Supplementary Table S7). The Kyoto Encyclopedia of Genes and Genomes pathway analysis showed that there was a strong connection in two areas: how cytokine interaction with their receptors (*P* = 0.0029) and PI3-AKT signaling pathway (*P* = 0.009) (Supplementary Table S8), highlighting the importance of inflammation and cell survival.

The protein-protein interaction (PPI) network demonstrated significant connectivity among proteins linked with glaucoma, forming nine distinct functional clusters (Fig. 2d; Supplementary Tables S9, S10). The largest group was related to organizing the extracellular matrix, which included 13 out of 180 proteins (FDR = 3.32 × 10^−6^), with important proteins like TIMP1, LTBP2, ELN, and MMP12. The second major cluster represented PI3K signaling pathways (9/154 proteins, FDR = 0.004), encompassing critical proteins such as HGF, GDF15, and NTRK2. Cross-validation using weighted gene co-expression network analysis (WGCNA) found 13 hub proteins with high connectivity (Supplementary Fig. S2; Supplementary Tables S11–S13). Importantly, 11 out of the 13 WGCNA hub proteins (84.6%) were connected in the PPI network, with strong support for key proteins like SCARB2 (PPI degree = 42), TGFBR2 (degree = 24), GDF15 (degree = 24), and EPHA2 (degree = 20), which strongly supports the importance of our network-based findings (Fig. 2e).

### Causal Protein Identification

**Figure 3.**
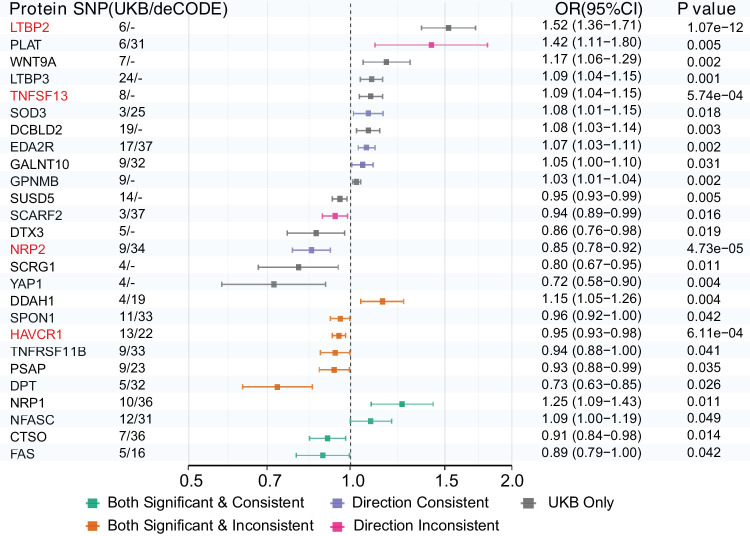
**MR analysis of glaucoma-associated proteins.** Forest plot displaying causal relationships between plasma proteins and glaucoma risk using two-sample MR. Results are shown for proteins with available instrumental variables from both UK Biobank (UKB) and deCODE genetics datasets. The left column shows protein names with the number of SNPs used as instruments for UKB/deCODE analyses, respectively. OR with 95% CIs are presented graphically in the forest plot and numerically on the right, along with corresponding *P* values. The *vertical dashed line* represents OR = 1.0 (no effect). Color coding indicates cross-platform validation situations. Proteins highlighted in *red text* (LTBP2, TNFSF13, NRP2, HAVCR1) represent the four proteins with the strongest evidence for causal relationships with glaucoma risk after multiple testing corrections.

Although functional enrichment revealed biological pathways related to glaucoma-associated proteins, differentiating causal determinants from downstream markers requires genetic proof. Consequently, we used Mendelian randomization (MR) to evaluate causal relationships using cis-pQTL as instrumental factors. Out of the 136 glaucoma-associated proteins, 109 had appropriate instrumental variables available and were subjected to MR analysis (Supplementary Table S14). Four proteins demonstrated strong causal links after FDR correction: LTBP2 (OR = 1.52 [1.36, 1.71]; *P* = 1.07 × 10^−^^12^), NRP2 (OR = 0.85 [0.78, 0.92]; *P* = 4.73 × 10^−^^5^), TNFSF13 (OR = 1.09 [1.04, 1.15]; *P* = 0.00057), and HAVCR1 (OR = 0.95 [0.93, 0.98]; *P* = 0.00061) (Supplementary Table S15). The detailed sensitivity tests showed that these results are trustworthy with no signs of heterogeneity or horizontal pleiotropy (Supplementary Tables S16, S17). Reverse MR analysis indicated no causal effect of glaucoma on protein levels (Supplementary Table S18). Cross-platform validation using deCODE data offers independent replication,^5^ identifying consistent causal effects for several proteins, including NRP1 (OR = 1.25 [1.09,1.43], *P* = 0.011), NFASC (OR = 1.09 [1.00, 1.19], *P* = 0.042), CTSO (OR = 0.91 [0.84, 0.98], *P* = 0.014), and FAS (OR = 0.89 [0.79, 1.00], *P* = 0.042) (Fig. 3; Supplementary Table S19), thereby strengthening the evidence for protein-mediated causal pathways in glaucoma pathogenesis.

### Temporal Dynamics of Glaucoma-Associated Proteins

**Figure 4.**
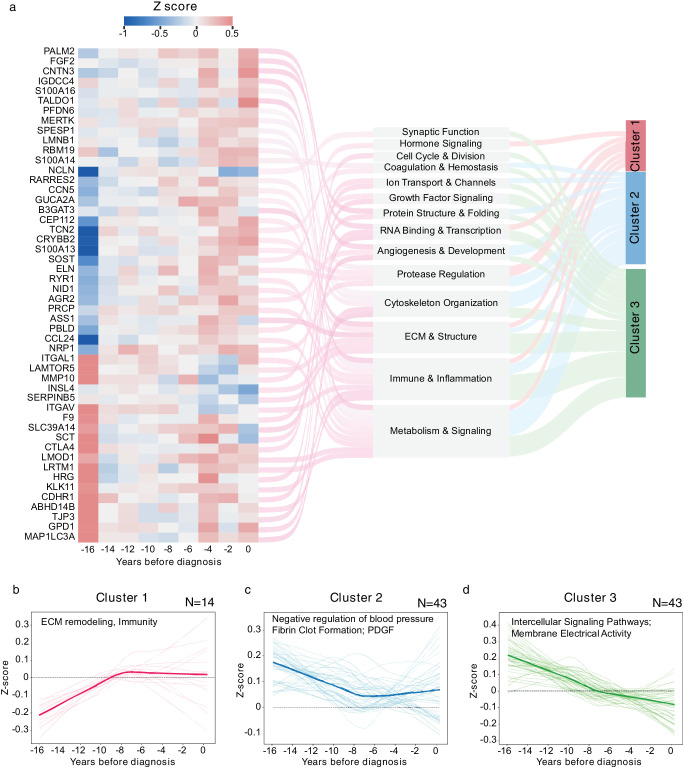
**Temporal dynamics of glaucoma-associated proteins preceding diagnosis.** **(a)** Z-score changes of glaucoma-associated proteins. The trajectories were estimated by LOESS. Heat represents absolute Z-score greater than 0.3. Proteins were mapped to functional categories shown in the middle. Hierarchical clustering identified three distinct temporal trajectory pattern (cluster 1–3, indicated by colored bars on the right). **(b–d)** Protein trajectories of identified clusters. Clusters were grouped using unsupervised hierarchical clustering, with the *bold lines* represents the average trajectory in each cluster.

We then used normalized *Z*-scores and LOESS regression to analyze the 136 proteins linked to glaucoma over the time leading up to diagnosis. This enabled us to delineate the temporal progression of the identified biological pathway. We detected early indicators of protein dysregulation 12 to 15 years before the disease's diagnosis. MAP1LC3A, MMP10, and LAMTOR5 were the initial proteins to demonstrate abnormalities (|*Z*-score| ≧ 0.3) (Fig. 4a; Supplementary Table S20). Hierarchical clustering identified three distinct trajectory patterns (Figs. 4b–d; Supplementary Table S21): Cluster 1 (*n* = 14) showed a steady rise in proteins related to the extracellular matrix and immune response, especially important components of the PPI network like TIMP1 and MMP12. Cluster 2 (*n* = 43) demonstrated an initial decrease over approximately 16 years, followed by stabilization eight to 10 years before diagnosis, emphasizing proteins involved in blood pressure regulation, fibrin clot formation, and platelet-derived growth factor signaling. Cluster 3 (*n* = 43) displayed a continuous decline, concentrating on intercellular signaling proteins, particularly the MR-validated causal protein GDF15. The changes over time of the MR-validated causative proteins matched their functions: LTBP2, which is part of the extracellular matrix, showed an upward trend like Cluster 1, whereas NRP2, which is involved in signaling, demonstrated a downward trend like Cluster 3. This reinforces the biological validity of our causal assumptions.

### Time-Stratified Predictive Modeling

**Figure 5.**
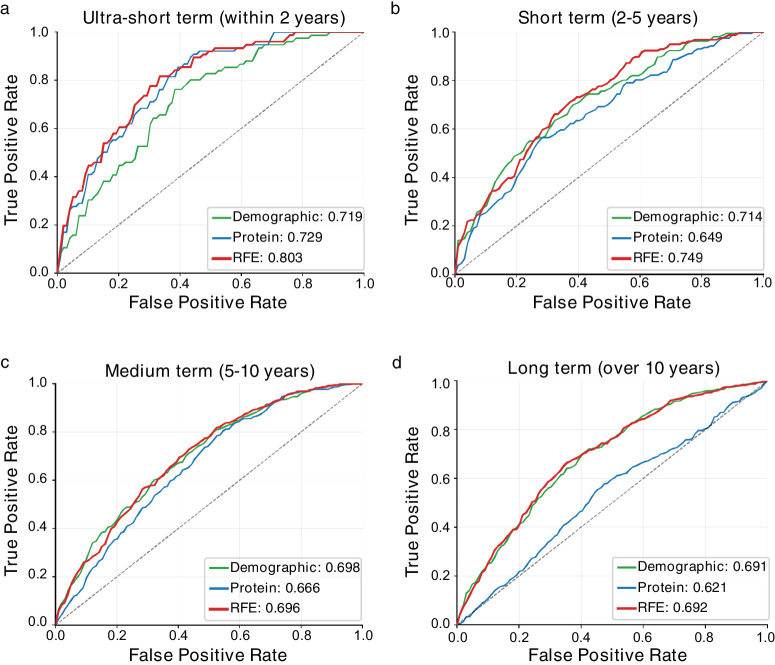
**ROC curves for future glaucoma prediction.** ROC curves comparing predicted performance across four temporal prediction windows: Ultra-short-term prediction (within two years) **(a)**, short-term prediction (2–5 years) **(b)**, medium-term prediction (5–10 years) **(c)**, and long-term prediction (over 10 years) **(d)**.

We developed time-stratified predictive models for four windows: ultra-short term (0–2 years), short term (2–5 years), medium term (5–10 years), and long term (beyond 10 years). Feature importance analysis revealed temporal shifts in predictive signature (Supplementary Table S22). Although age remained the top predictor across time frames, protein contributions varied markedly: ultra-short-term models relied on LILRB5, EDA2R, and GUCA2A, whereas medium-term prediction was dominated by NEFL (7.09), GDF15 (7.49), and ELN (6.2), reflecting evolving pathophysiological processes. Recursive feature elimination achieved remarkable performance with clear temporal stratification (Supplementary Table S23). Ultra-short-term prediction reached an AUC of 0.803 (0.602, 0.837) (Fig. 5a). Short-term achieved AUC = 0.748 (0.668, 0.829) (Fig. 5b), whereas medium-term and long-term models showed diminished discriminative ability (AUC = 0.696 and 0.692) with minimal improvements over conventional approaches (Figs. 5c-d). DeLong tests confirmed significant performance differences between time frame (*P* < 0.001 for ultra-short vs. Others, Supplementary Table S24). For comparison with clinical parameters to evaluate the clinical relevance of our proteomic model, we performed a sensitivity analysis in a subset of participants (*N* = 57 in the ultra-short-term window) who had valid baseline IOP measurements (Supplementary Table S31). In this sub-analysis, the proteomic prediction model achieved an AUC of 0.824, numerically outperforming the standard clinical model incorporating age, sex, and IOP (AUC = 0.758). Furthermore, adding IOP to the proteomic model did not significantly improve performance (combined AUC = 0.784), suggesting that the identified plasma biomarkers provide predictive information largely independent of ocular tension.

### Associations With Ophthalmic Parameters

**Figure 6.**
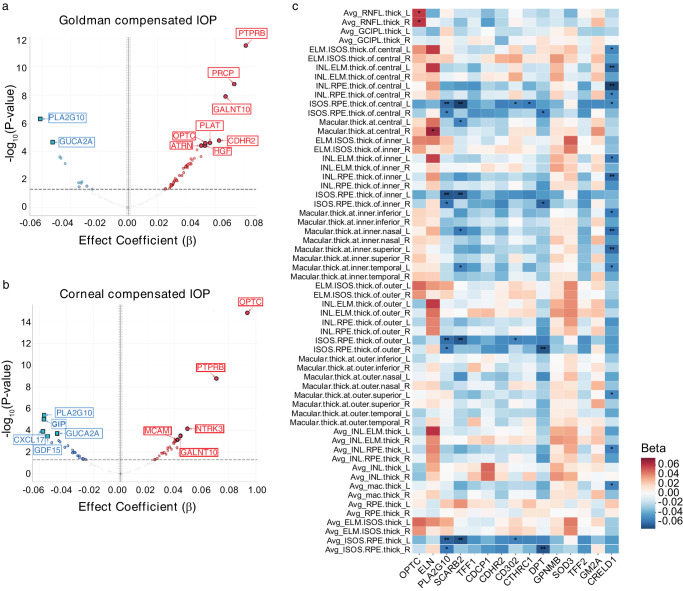
**Association between glaucoma-associated protein and ocular parameters. (a)** Volcano plot showing the HRs (x-axis) and statistical significance (−log10 of two-sided *P* value, y-axis) of the associations of glaucoma-associated proteins and Goldman-compensated IOP. **(b)** Volcano plot represents the analysis on corneal-compensated IOP. **(c**) Heatmap showing comprehensive associations between glaucoma-associated proteins (x-axis) and various retinal thickness measurements (y-axis) obtained from OCT. The color scale represents beta coefficients ranging from negative to positive (−0.06 to 0.06), suggesting their potential roles in retinal structural changes associated with glaucoma.

Regression analyses examined relationships between glaucoma-associated proteins and retinal imaging parameters. For Goldman-compensated IOP, PTPRB showed the strongest positive association (β = 0.081, *P* = 3.08 × 10^−^^12^), followed by PRCP (β = 0.073, *P* = 1.69 × 10^−^^9^) and GALNT10 (β = 0.067, *P* = 1.26 × 10^−^^8^), whereas PLA2G10 and GUCA2A showed negative associations (Fig. 6a; Supplementary Table S25). For corneal compensated IOP, OPTC exhibited the strongest association (β = 0.094, *P* = 1.48 × 10^−^^15^) (Fig. 6b; Supplementary Table S26). Analysis of segmented retinal layer thicknesses (including RNFL, GCIPL, INL, and photoreceptor layers) revealed proteins with widespread associations across multiple regions, including OPTC, PLA2G10, SCARB2, and CRELD1 (Fig. 6c; Supplementary Table S27).

### Drug Target Enrichment Analysis

Four genes related to glaucoma (NRP2, TNFSF13B, LTBP2, and HAVCR1) were identified as drug targets for 17 different treatments across nine categories (Supplementary Table S28). NRP2 was identified as a druggable target with five existing compounds, although current anti-VEGF agents that inhibit NRP2 are contraindicated for primary glaucoma prevention given our finding that reduced NRP2 levels increase risk; TNFSF13B to four compounds, including Belimumab; LTBP2 to five TGF-β pathway modulators; and HAVCR1 to three experimental immune checkpoint agents. The disease categories that had a higher presence of these genes included eye and surrounding areas, muscles and bones, tumors, and diseases of the urinary system (OR = 9.00, FDR = 0.145 for each category), with a moderate presence in nervous and respiratory system diseases (OR = 6.33, FDR = 0.276), but none were statistically significant after adjustments. LTBP2 and TNFSF13 represent more immediate therapeutic opportunities, with LTBP2 linked to TGF-β pathway modulators and TNFSF13 to Belimumab, aligning with the pathophysiological mechanisms identified in our temporal analysis.

## Discussion

### Summary of the Results

In this prospective population-based cohort study of 45,850 UK Biobank participants followed up for a median of 16.26 years, we identified 136 plasma proteins associated with clinical diagnosis, with EDA2R showing the strongest association (HR = 1.21; 95% CI, 1.16–1.25; *P* = 2.99 × 10^−^^17^). MR confirmed causal relationships for four proteins: LTBP2 (OR = 1.52; 95% CI, 1.36–1.71; *P* = 1.07 × 10^−^^12^), NRP2 (OR = 0.85; 95% CI, 0.78–0.92; *P* = 4.73 × 10^−^^5^), TNFSF13, and HAVCR1. A proteomic risk model achieved a concordance of 0.803 (95% CI, 0.772–0.837) for near-term prediction, improving from 0.700 using demographic factors alone.

### Relationship With the Literature

Our proteomic approach complements recent genetic risk stratification efforts in glaucoma. Although de Vries et al.^15^ achieved a C-statistic of 0.82 using polygenic risk scores combined with IOP and family history, our protein-only model achieved 0.803 without these parameters. This suggests that plasma proteins capture both genetic predisposition and acquired risk factors independently.

Contemporary diagnostic approaches remain limited to symptomatic disease diagnosis.^16^ Although artificial intelligence–enhanced methods have achieved an accuracy of 91% to 93% by integrating visual fields with clinical data.^17^^,^^18^ Proteomic investigations have identified multiple high-efficacy biomarkers, including serum apolipoprotein A4 (APOA4), which exhibits a diagnostic accuracy of 97%.^19^ However, proteomic studies often examined limited cohorts of patients (10–60 patients) at a singular time point, failing to capture the temporal variations in biomarker as the disease advances. Our longitudinal analysis of 45850 participants reveals the temporal evolution of proteomic changes, demonstrating genuine predictive rather than diagnostic capability.

Furthermore, our findings offer distinct insights compared to recent broad-proteomic studies.^11^^,^^20^ Although these studies provide a valuable “breadth” of the proteome across multiple diseases, our study focuses on “depth” specifically for glaucoma. First, unlike general scans that use baseline measurements to predict overall incidence, our time-stratified analysis reveals the dynamic temporal evolution of proteins. We identified that specific markers are elevated specifically in the 12 to 15 years preclinical window, a nuance missed by snapshot analyses. Second, broad pipelines often apply automated MR to thousands of traits. In contrast, our study used manually curated MR instruments specific to ocular physiology, allowing us to rigorously distinguish between causal drivers and downstream markers of optic nerve damage. Finally, by adjusting for ocular-specific covariates, we isolate systemic proteomic risks that are independent of local mechanical factors, providing a more precise risk profile than general phenome-wide scans.

The proteins we identified validate and extend genetic discoveries. LTBP2, previously identified through genetic studies as causative for familial glaucoma, showed the strongest causal evidence in our population-based analysis.^21^ This convergence of genetic and proteomic evidence strongly supports LTBP2 as a central mediator in glaucoma pathogenesis, bridging inherited and acquired forms of the disease through common mechanistic pathways involving extracellular matrix dysregulation and enhanced pressure sensitivity. Conversely, NRP2 downregulation (OR = 0.85) challenges current paradigms. Interestingly, our cross-platform validation identified its homolog, NRP1, as a causal risk factor (OR = 1.25), suggesting a divergent role for these neuropilins in glaucoma. Although NRP1 and NRP2 share structural homology, they likely influence intraocular pressure via distinct mechanisms. NRP1 is known to activate latent TGF-β, a cytokine central to trabecular meshwork fibrosis and increased outflow resistance.^22^ In contrast, NRP2 is essential for maintaining the lymphatic-like phenotype of Schlemm's canal, which is critical for efficient aqueous humor drainage.^23^ Thus the opposing causal effects observed in our study highlight the complexity of VEGF/Semaphorin signaling in the eye. Specifically, NRP1 appears to promote risk via fibrosis, whereas NRP2 offers protection through drainage maintenance. This implies that therapeutic strategies should specifically enhance NRP2 or inhibit NRP1, rather than broadly targeting the neuropilin family.

Novel associations warrant attention. EDA2R suggests epithelial-mesenchymal transition pathways contribute to glaucoma pathogenesis. GDF15’s consistent reduction beginning 12 to 15 years before diagnosis, highlighting its potential as a marker for systemic stress response. NEFL, a classical neurodegeneration marker,^24^ indicates early axonal dysfunction detectable peripherally years before clinical manifestation.

### Clinical Implications

Our temporal analysis revealed three therapeutic windows: early extracellular matrix distribution (12–15 years), intermediate metabolic reprogramming (8–12 years), and late neurodegeneration (0–5 years). This staged progression parallels OHTS and EMGT findings demonstrating 60% risk reduction with early intervention. Our 15-year window could enable even earlier intervention, potentially achieving greater risk reduction. Clinicians may question how this proteomic approach compares to established diagnostics like OCT RNFL thinning or visual field perimetry. It is important to distinguish their roles: OCT and perimetry are diagnostic tools for detecting existing structural and functional damage. In contrast, our findings define a “preclinical window” of proteomics that serves not to replace OCT but to act as an early risk stratification filter, identifying high-risk individuals who warrant earlier and more frequent ophthalmologic surveillance before irreversible damage occurs.

Drug target analysis reveals immediate translation opportunities. LTBP2-targeting TGF-β modulators show promise given strong causal evidence and established role of TGF-β dysregulation in trabecular meshwork fibrosis.^25^ TNFSF13’s association with Belimumab suggests novel immune modulation strategies. However, NRP2’s protective role contradicts current anti-VEGF approaches for primary glaucoma prevention, necessitating development of enhancement strategies.

### Strengths and Limitations

The population-based design with 16-year follow-up represents the primary strength, capturing the full disease trajectory unlike previous proteomic studies. Mendelian randomization distinguishes causal relationships from associations, providing confidence for therapeutic targeting. The inclusion of temporal analysis revealed evolving proteomic signatures, with different proteins dominating prediction at various time windows.

To rigorously address the potential of reverse causality, we conducted sensitivity analyses excluding participants diagnosed within 5 years of baseline (Supplementary Table S30). Key associations remained robust; notably, EDA2R, GDF15, and NEFL retained high statistical significance (*P* < 0.001) even in this strictly filtered subset. This stability, coupled with our observation of trajectory divergence up to 15 years prior to diagnosis, reinforces that these proteomic signals reflect early pathogenesis or long-term susceptibility rather than markers of undiagnosed, prevalent disease.

Several limitations merit consideration. First, our glaucoma definition relies on diagnostic codes and self-reports rather than clinical adjudication with visual field or optic disc parameters (e.g., vertical cup-to-disc ratio). According to current clinical guidelines, standard automated perimetry (VF testing) remains the functional gold standard for diagnosing and staging glaucoma, as it directly assesses the loss of peripheral vision characteristic of optic neuropathy. The absence of longitudinal VF data in our definition means we may miss early functional progression or misclassify glaucoma suspects who have not yet developed definitive field loss. Relatedly, this definition pools all glaucoma subtypes into a single outcome. We acknowledge that distinct subtypes (e.g., primary open-angle vs. angle-closure glaucoma) possess unique biological mechanisms. However, given that POAG accounts for the vast majority of cases in European populations, our findings likely reflect POAG-dominant biology. Furthermore, previous genetic validation has shown a high correlation between this composite phenotype and clinically defined POAG, suggesting they share a nearly identical biological architecture. Any inclusion of non-POAG cases likely introduces non-differential misclassification, biasing our results toward the null rather than creating spurious associations.

Second, a major limitation is the lack of an external validation cohort, particularly one with racial diversity. Our study population was predominantly of European ancestry (93.1%), which restricts the generalizability of our findings. This is critical given that glaucoma prevalence and severity vary substantially across racial groups. For instance, individuals of African descent often experience an earlier onset and more aggressive disease course. Consequently, the proteomic signatures identified here may not fully capture the pathophysiological mechanisms relevant to other high-risk groups. Future studies must prioritize validation in diverse cohorts to ensure equitable clinical utility.

Third, the Olink platform covers a subset of the plasma proteome, and a single baseline measurement cannot capture the dynamic fluctuations. Fourth, although we adjusted for key technical and demographic covariates, certain ocular and systemic parameters were not included in the primary model due to data availability. Specifically, baseline intraocular pressure and central corneal thickness were available for only a subset of participants. To address this, we conducted a sensitivity analysis in the sub-cohort with valid IOP measurements (*n* = 9442) (Supplementary Table S32). Crucially, the effect sizes for our top candidate proteins (e.g., EDA2R, LTBP2) remained robust after additionally adjusting for IOP, suggesting these associations are largely independent of ocular tension and may reflect direct neurodegenerative or extracellular matrix remodeling pathways. Similarly, adjusting for systolic blood pressure in a sensitivity analysis did not materially alter our findings, ruling out systemic hypertension as a primary confounder. Finally, although we observed a statistical association with alcohol consumption, this should be interpreted with caution. Given that alcohol is not an established independent risk factor for glaucoma, this finding likely reflects residual confounding by unmeasured socioeconomic or lifestyle variables rather than a direct pathophysiological mechanism.

#### Further Consideration

Cost-benefit analyses should evaluate whether proteomic screening could be economically viable, particularly when combined with polygenic risk scores. Integration could yield superior models, as proteins capture both inherited susceptibility and acquired risk factors. Validation in diverse populations and glaucoma subtypes remains essential for clinical translation.

## Conclusions

This prospective study of 45,850 participants reveals that plasma proteomic dysregulation commences 12 to 15 years before glaucoma diagnosis, substantially earlier than previously recognized. Mendelian randomization identified LTBP2, NRP2, TNFSF13, and HAVCR1 as causal proteins, implicating extracellular matrix remodeling and immune dysregulation in disease pathogenesis. The distinct temporal trajectories and strong predictive performance (AUC = 0.803 for near-term prediction) highlight the mechanistic insights and future translational potential of plasma proteomics. Although these findings suggest a pathway toward proactive, protein-guided risk stratification, substantial additional validation, health-economic analysis, and interventional studies are required to determine the feasibility of integrating such approaches into routine glaucoma care.

## Supplementary Material

Supplement 1

Supplement 2
