# Supplementary material for: Plasma Proteomic Dynamics Preceding Glaucoma Reveal a 15-Year Pre-Diagnostic Window: Causal Insights and Predictive Utility in 45,850 Participants
Source: Invest Ophthalmol Vis Sci. 2026 Mar 6;67(3):14. doi: 10.1167/iovs.67.3.14 (PMC12974536; doi:10.1167/iovs.67.3.14)
Supplement: Supplement 1 [file iovs-67-3-14_s001.docx]

Supplementary Information For

**Large-scale plasma proteomic profiling identifies early biomarkers and establishes predictive model for glaucoma**

**Supplementary Figure 1. Time-stratified analysis of glaucoma-associated proteins and functional enrichment. a.**Volcano plot displaying the association between plasma protein and glaucoma risk within 10 years. The x-axis represents hazard ratio (HR) and the y-axis shows -log10(P-values). Dash lines indicate HR=1.0 (vertical) and statistical significance threshold (horizontal). Key proteins with strongest associations are labeled. **b**. GO enrichment analysis showing the biological processes significantly associated with <10 years glaucoma risk. **c**. Analysis of glaucoma incidence beyong 10 years. D Enrichment analysis for long-term associations.

**Supplementary Figure 2.** **Weighted Gene Co-expression Network Analysis (WGCNA) of glaucoma-associated proteins. a.** Network topology for the soft-thresholding power selection. **b.** Hierarchical cluster of glaucoma associated proteins based co-expression model. **c.** Hub gene interaction network.
